# Effect of Nano Silicon Dioxide Coating Films on the Quality Characteristics of Fresh-Cut Cantaloupe

**DOI:** 10.3390/membranes11020140

**Published:** 2021-02-17

**Authors:** Rokayya Sami, Manal Almatrafi, Abeer Elhakem, Mona Alharbi, Nada Benajiba, Mahmoud Helal

**Affiliations:** 1 Department of Food Science and Nutrition, College of Sciences, Taif University, Box P.O. 11099, Taif 21944, Saudi Arabia; manal.almatrafi@uconn.edu; 2 Department of Biology, College of Science and humanities in Al-Kharj, Prince Sattam Bin Abdulaziz University, Al-Kharj 11942, Saudi Arabia; a.elhakem@psau.edu.sa (A.E.); mh.alharbi@psau.edu.sa (M.A.); 3 Department of Basic Health Sciences, Deanship of Preparatory Year, Princess Nourah Bint Abdulrahman University, P.O. Box 84428, Riyadh 11671, Saudi Arabia; nabenajiba@pnu.edu.sa; 4 Department of Mechanical Engineering, Faculty of Engineering, Taif University, Box P.O. 11099, Taif 21944, Saudi Arabia; helal.mo@tu.edu.sa

**Keywords:** chitosan, nano-silicon dioxide, shelf-life, cantaloupe, microbial activity

## Abstract

The prime objective of the research was to explore the coating effects of chitosan and nano-silicon dioxide with nisin as an antimicrobial agent on physicochemical properties, microbiological stability, and sensorial quality changes during the storage at 4 °C. The combination of nano-material and chitosan in addition to nisin was effective for reducing the postharvest attributes of fresh-cut cantaloupes in addition to the highest score in sensory evaluation. Chitosan coating treatment enhanced the microbiological quality 2.50 log CFU/g and 1.87 log CFU/g for aerobic counts and mold/yeasts populations, respectively. In a word, the combination of chitosan/nano-silica/nisin treatment was the best condition for fresh-cut cantaloupe shelf life extension by maintaining color, vitamin C 22.29 mg/100g, peroxidase activity 8.06 U/min.g, and other microbiological tests up to storage time of 8 days.

## 1. Introduction

Fresh-cut products make easy access to meet the demands of consumers for vegetables and fruits [1]. Cantaloupe (*Cucumis melo* L.) is an example, requiring preparation before eating due to its too large size [2]. Fruit quality properties such as color, texture, and microbial quality are the top priority because of consumption and fetching commercial profits [3]. Cantaloupe is an excellent vitamin C and β-carotene source; it also provides additional nutritional values of iron, potassium, and dietary fiber [4]. Some of the common and cost-effective methods are used for developing the quality of cantaloupe products includes the packaging by ultraviolet light [5,6], gamma radiation [7], chemical treatments [8], edible coatings [9], and multilayered edible coatings using nanotechnology [10]. This coating technique could be perfect for overcominge quality problems [11]. Chitosan is recognized due to its antimicrobial activity and hybrid film properties [12]. Nutrients, colorants, antioxidants, antimicrobials, and flavors can be included in the films and discharged in an organized manner [13]. Another food additive; nano-silicon dioxide, has been approved for safety rating which cannot be digestible by humans [14,15]. Earlier research works reported chitosan and nano-silicon dioxide coatings to reduce browning of jujube, papaya, longan, and apple fruits [16,17,18,19]. Nisin is defined as an antimicrobial peptide and approved by (FAO/WHO) as one of the common safe food additives for different types of foods [20].

Therefore, according to the advantage of eco-friendly and cost-effective coating techniques, the present work aimed to indicate the efficiency of chitosan/nano-silica/nisin coating to improve fresh-cut cantaloupe preservation quality at 4 °C of storage.

## 2. Materials and Methods

### 2.1. Materials

Chitosan deacetylation medium of 85% molecular weight, acetic acid, and glycerol were purchased from Jinde Haidebei Marine Biological Engineering Co., Ltd., Tangshan, China. Tetraorthosilicate (TEOS) as nano-SiO_2_ and Nisin were supplied by Caofeidian Taihongshengda New Material Co. (Tangshan, China). All other chemicals belong to the analytical grade. 

### 2.2. Fruits

A total number of 4 batches of cantaloupes melon were procured from a local commercial fruit store, washed with running water, peeled, dissected into 30 × 30 × 30 (mm) slices, and stored in a refrigerator at a storage temperature of 4 °C with 65% relative humidity. 

### 2.3. Preparation of Hybrid Coating Film

Chitosan (1%) was dissolved in 1% acetic acid and 0.5% glycerol. The solution was stirred 10 h at 300 rpm, centrifuged at 4 °C for 30 min to separate the supernatants and remove insoluble particles. The same amount of this solution was taken and 1% of TEOS was added in a 500 mL flask. Nisin was dissolved in a chitosan/nano-silica solution containing 0.02 mol/L hydrochloric acid. 

### 2.4. Treatment of Fresh-Cut Cantaloupe Fruit

The cut fruit pieces were distributed into four groups randomly as follows: control (deionized water); chitosan (CTS); chitosan/nano-silica (Nano/CTS) and chitosan/nano-silica/nisin (Nano/CTS/N). The fresh-cut cantaloupe fruit samples were dipped into different solutions for 5 min and allowed to be dry before chilling at 4 °C.

### 2.5. Shelf-Life Analysis

Fresh-cut cantaloupe samples were placed in polyethylene zipper bags, having a 0.02-millimeter thickness and kept at 4 °C. Physical, chemical, microbial, and sensorial parameters were carried out at different intervals of storage days 0, 2, 4, 6, and 8 in refrigeration. 

#### 2.5.1. Product Quality Parameters

##### Fluid Loss 

The fluid loss was measured according to the weights of juice and sample in the bag throughout the storage [2].

##### Colour Determination

The color was detected by a ZE-6000 Meter (Nippon, Japan). D65 light source and an 8 mm diameter measuring area were used [9].

#### 2.5.2. Physicochemical Quality Analysis

##### pH, Total Soluble Solids, Titratable Acidity, and Vitamin C Determinations

The pH value was measured at the ambient temperature (approximately 27 °C) from the cantaloupe juices by using a pH meter (Mettler Toledo Instruments Co., Shanghai, China) [21]. Total soluble solids content (TSS) was examined using a refractometer (Atago Pocket-refractometer PAL-BX/RI, Tokyo, Japan). Total acidity (TA) was detected by sodium hydroxide solution (0.1 M) titration [22], and results were calculated as the percent of citric acid. Finally, vitamin C (Vc) was detected by iodine titration, and results were calculated as milligrams/100 g of the sample [23].

##### Extraction of Malondialdehyde Content

A mass of selected tissue (3 g) of fresh-cut-cantaloupe fruits was blended with 10 mL of 10% trichloroacetic acid to detect malondialdehyde content (MDA) [24]. The supernatant was blended with 2 mL of 0.5% 2-thiobarbituric acid, boiled for 20 min at 95 °C, immediately cooled, and then the supernatant was taken in a 96-well plate. The absorbance was evaluated at 450, 532, and 600 nm. 

##### Polyphenol Oxidase Enzyme Activity Determination 

Polyphenol oxidase (PPO) activity: 5 mL of 0.2 mol/L with phosphate buffer (pH 7) was added to 1 mL 0.1 mol/L pyrocatechol solution and 1.95 mL 0.2 mol/L with phosphate buffer (pH 7) [19]. The absorbance increase was measured every 20 s within 6 min after the addition of cantaloupe extract at (410 nm). 

##### Peroxidase Enzyme Activity Determination 

Peroxidase (POD) activity: 5 mL of 0.2 mol/L with phosphate buffer (pH 7) was added to 0.15 mL 10 g/L guaiacol, 0.15 mL volume fraction 1% H_2_O_2_, 2.66 mL with phosphate buffer (pH 7), and the absorbance at (460 nm) was measured every 20 s within 6 min [24].

#### 2.5.3. Microbiological Analysis and Water Activity Determination

The analyses of the aerobic plate, yeast, and mold counts were carried out every 2 days until the storage period end [25]. Aerobic plate, yeasts, and molds performed using two Rose Bengal Mediums (RBM) (GB4789.15-2016) and (GB4789.2-2016) from Cell Bank (Biological Sciences, Shanghai, China). All the plates were incubated at 28 °C for 5 days. The obtained colonies were counted as log CFU (colony-forming units)/grams of cantaloupes at the incubation period ends. The water activity (Aw) was evaluated using a water activity meter (Aqualab, Decagon Devices, California, USA) [26].

#### 2.5.4. Sensory Analysis

Sensory analysis was achieved consisting of students, qualified and experienced staff in College of Sciences with a range of (21–35) years. The assessment was done on the last day of the storage time. Panelists were given uniform amounts of each coating treatment to evaluate (color, odor, texture, flavor, and overall quality). Assessments were estimated according to the previous report of fresh-cut cantaloupe sensory determination [27].

### 2.6. Statistical Analysis

All the recorded data was recorded in triplicate and applied by using the SPSS Version 20.0 (SPSS Inc., U.S.A.). Significant differences were detected by Duncan’s multiple tests.

## 3. Results and Discussion

### 3.1. Effect of Coating Treatment on Fluid Loss and Colour Index 

**Figure 1:**
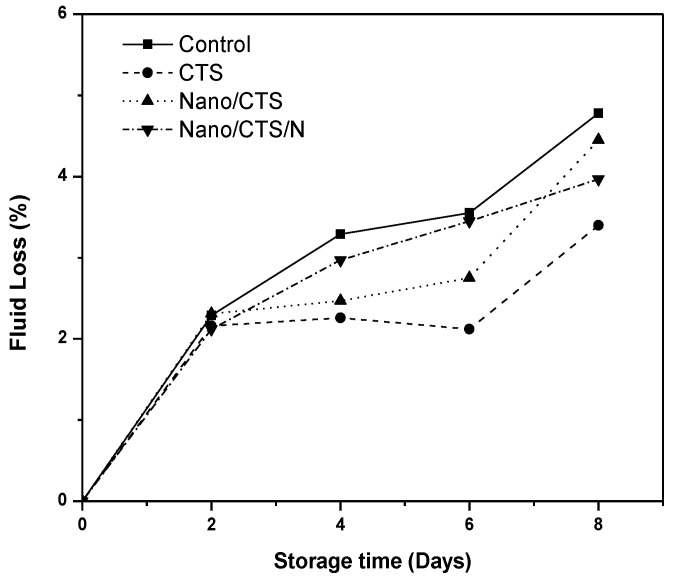
Effects of coating treatments on fluid loss for cantaloupes during storage at 4 °C for 8 days. Data are mean ± SD, *n* = 3.

Figure 1 shows fluid loss during chilled storage for fresh-cut cantaloupes. All samples lost their weight gradually due to moisture evaporation and respiration [28]. The fluid loss reached ~4% at the end of the whole storage period. The same results were also reported [29]. The cantaloupe’s color became significantly darker after the 2nd day of the storage for all the treatments except for Nano/CTS/N treatment, whose *L* values decreasing from 38.53 to 23.42 (Table 1). Falade et al. [30] reported that enzymatic processes and weight loss may increase the pigment as β-carotene in watermelon. Zhang et al. [31] detected the same results for color determination. For *a* and *b* values, no differences were detected among all treatments, proposing that coating had no clear effects on the cantaloupe appearance. The results recommend that (Nano/CTS/N) treatment can help in maintaining cantaloupes color during the whole storage.

### 3.2. Effect of Coating Treatment on pH, TSS, TA, and Vc Contents

**Figure 2:**
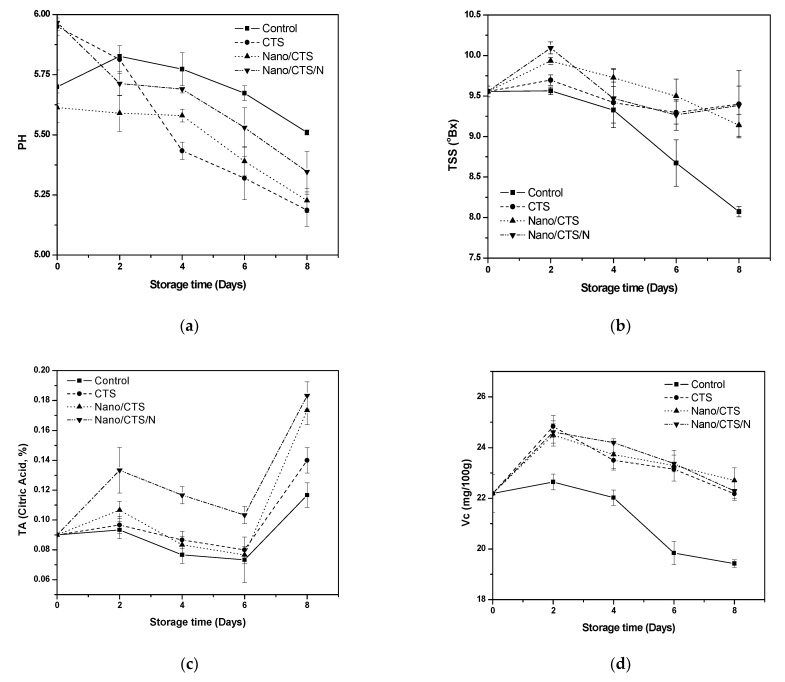
Effects of coating treatments on pH (**a**), total soluble solids (TSS) (**b**), total (TA) (**c**), and retention (Vc) (**d**) contents of cantaloupe fruit; data are mean ± SD, *n* = 3.

The application of the different coating treatments affected the chemical parameters of fresh-cut cantaloupes. The pH was in-between 5.67–5.99 during the inertial storage (Figure 2a). CTS treatment had a slightly lower pH value than the other treatments. All results were within the previous range of cantaloupe (5.5–6.5) [2]. All treatments had Brix values TSS in-between ~9.56 during the inertial storage and reached 8.07 to 9.4 by the end of the storage time (Figure 2b). Kaushlendra et al. [32], mentioned that cantaloupe sugar contents cannot be changed obviously as it is a non-climacteric fruit. 

Total (TA) of all fresh samples had ~0.09 during the inertial storage. On the 8th day, TA values increased although independent of CTS and nano-materials in the different coating treatments (Figure 2c). The coating was operative in the ripening process delay for fresh-cut cantaloupe. Similar TA values were found [26]. The coating helped retention (Vc) in all the treatments, which showed a little increase from 22.19 to 22.71 mg/100g (Figure 2d). Similar results were detected as vitamin C content increased until the 8th day then decreased significantly during the evaluation due to the respiration decreased rate and oxygen reduced [29].

### 3.3. Effect of Coating Treatment on MDA Content 

**Figure 3:**
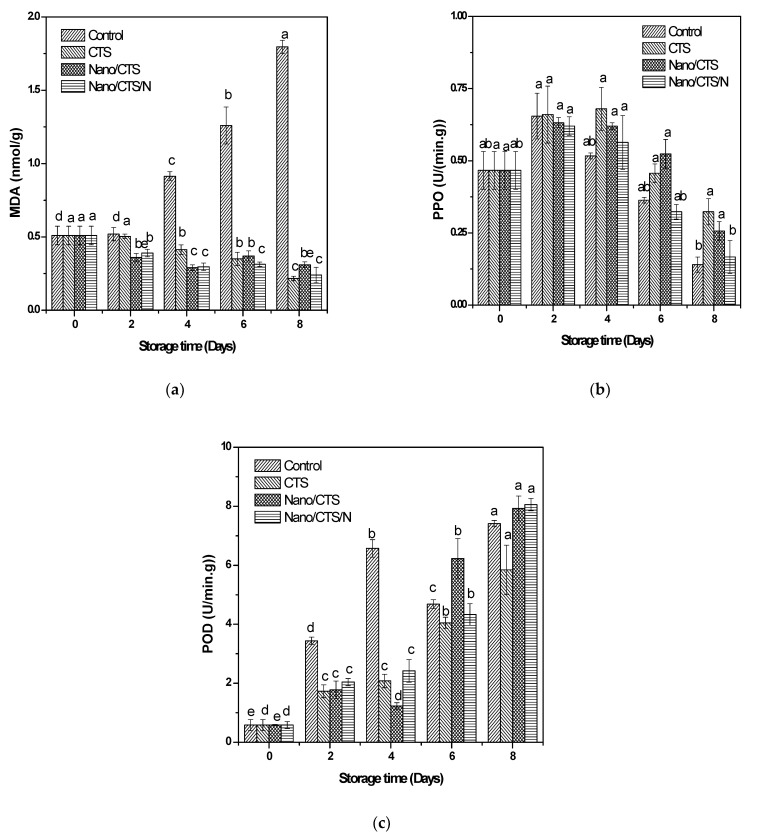
Effects of coating treatments on malondialdehyde content (MDA) (**a**), polyphenol oxidase (PPO) (**b**) and peroxidase (POD) (**c**) activities of cantaloupe fruit; ^a;b;c;d;e^ mean significant differences between treatments at *p* ≥ 0.05.

MDA is often used as a sign of fruit damage progress due to the structural integrity and cell membrane lipid peroxide levels [33]. As shown in (Figure 3a), a constant increase was detected for the control samples, while the treatment of nano-material delayed MDA increases. CTS treatment had 0.22 nmol/g, which was lower than the other samples by the end of the storage period. It suggested that all nano-treatments can inhibit the peroxidation of lipids during storage.

### 3.4. Effect of Coating Treatment on PPO and POD Enzyme Activities

As shown in (Figure 3b), PPO enzyme activities ascended radically in all the treatments during the storage which reached the maximum 0.14 U/min.g for control on the 8th day. CTS treatment reached 0.68 U/min.g on the 4th day, comparing with Nano/CTS 0.52 U/min.g on the 6th day, while Nano/CTS/N recorded the lowest PPO activity by the end of the whole storage. These results were in agreement with the PPO observation, where it is reported that the maintenance of POD activity in Nano/CST and Nano/CST/N treatments increased due to the presence of consistent abiotic stress in the cantaloupe pieces during storage [34]. PPO values detected in grape and strawberry preserved by nano-materials and CTS also showed less damage than the control [24,35].

CTS treatment had the lowest POD enzyme activity as (5.84 U/min.g) comparing the control (Figure 3c). The increase of POD enzyme activity in the coated cantaloupe fruits could reflect tissue damage progress during storage [36].

### 3.5. Effect of Coating Treatment on Microbiological Quality 

**Figure 4:**
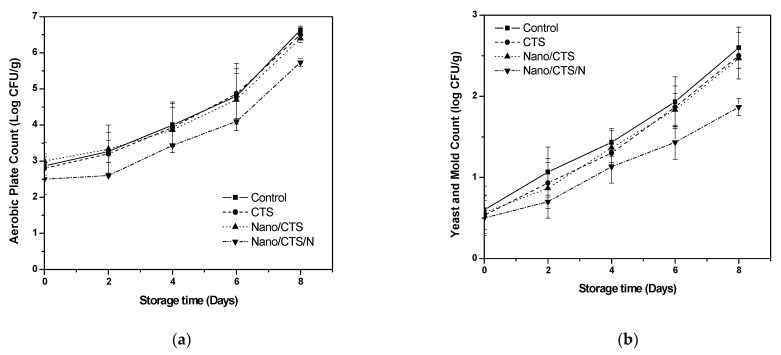

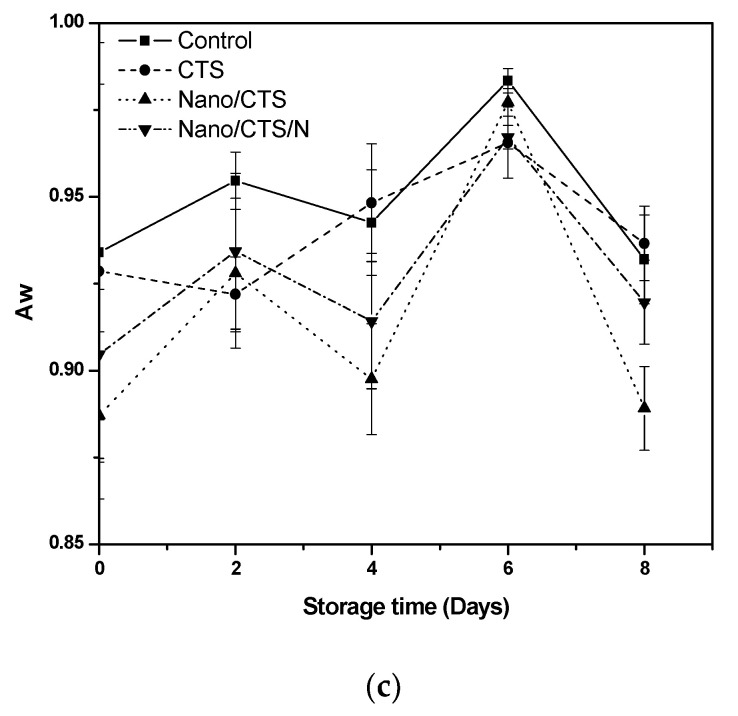
Effects of coating treatments on microbial quality, (**a**) total aerobic plate count, (**b**) yeast and mold counts, and (**c**) water activity of cantaloupe fruit; data are mean ± SD, *n* = 3.

The development of microorganisms is shown in (Figure 4a). The internal aerobic microorganism counts were significantly higher in the control sample as (2.87 log CFU/g) and Nano/CTS (3.00 log CFU/g) compared with CTS (2.80 log CFU/g) and Nano/CTS/N (2.50 log CFU/g), respectively. A gradual increase was detected in the microbial growth for all the treatments during the storage. However, by the end of the storage, the control recorded (6.63 log CFU/g), followed by Nano/CTS and CTS treatments, whose microbial level had a little similarity (*p* ≥ 0.05) (ranging from 6.50 to 6.40 log CFU/g). Finally, the cantaloupes treated with Nano/CTS/N presented the lowest microbial counts (5.73 log CFU/g) at the end of the storage time [37]. 

The presence of mold and yeasts is presented in (Figure 4b). Nano/CTS/N treatment reduced the growth to (1.87 log CFU/g), followed by Nano/CTS, whose microbial level was (2.47 log CFU/g) on the 8th day of storage, respectively. Finally, the control recorded the highest counts as (2.60 log CFU/g). CTS treatment could decrease the microbial growth of yeasts and molds, which was in agreement with Syahidah et al. [38]. Chong et al. (2015) [22] suggested that the positive protonated (NH^3+^) in the (C-2) of the polysaccharide can react with phosphoryl groups in the cell membrane. Similar reports have been approved [12,39]. Aw is responsible for the cell wall water holding capacity of cantaloupe tissue. The (Aw) of untreated cantaloupes ~0.93 was different compared to all the treated ones with nano-material treatments (0.88–0.91) as shown in (Figure 4c).

### 3.6. Sensory Evaluation of Coating Treatment

**Figure 5:**
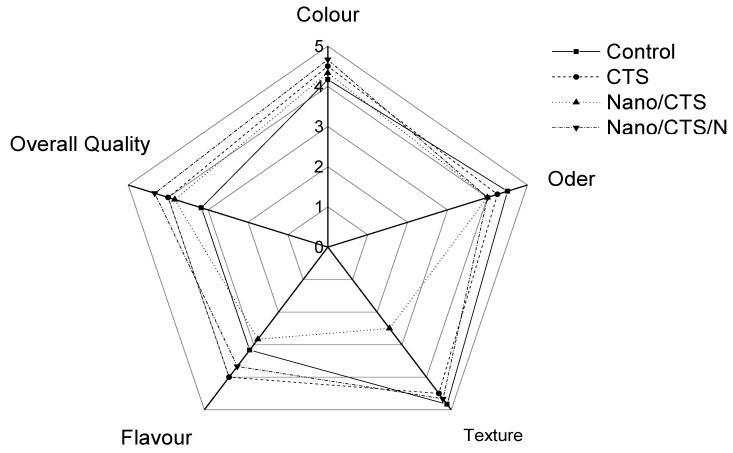
Sensory evaluation of fresh-cut cantaloupe fruits.

The sensorial properties made by different coating treatments such as color, odor, flavor, texture, and overall quality are shown in (Figure 5). Control and Nano/CTS sample treatments showed the least acceptance score in a comparison with the other sample treatments; while Nano/CTS/N treatment sample gave the highest score. It may be as a result of higher microbial activities [37]. According to the results, it can be established that using the Nano/CTS/N condition was the most acceptable to be used in cantaloupe preservation.

## 4. Conclusions

The modified chitosan/nano-silica/nisin hybrid films with non-destructive coating were applied for cantaloupe preservation during chilled storage to extend the shelf life. The retrieved investigated data depicted that the chitosan/nano-silica with the addition of nisin coating treatment was the most effective by forming semi-films against aerobic microorganisms, yeasts, molds, enzyme activities, and sensory evaluation.

Besides, artificial and natural chitosan-inorganic films can offer intriguing opportunities for nanotechnology application research and also provide new techniques in bringing solutions for the bioprocessing industry and fruit preservation. 

## Figures and Tables

**Table 1:** Changes in L*-values, a*-values, and b*-values of cantaloupe during storage.

	Control	CTS	Nano/CTS	Nano/CTS/N
L*-value				
0	^A^ 38.53 ± 0.45 ^a^	^B^ 37.06 ± 0.39 ^a^	^C^ 26.31 ± 0.38 ^b^	^C^ 26.20 ± 1.34 ^bc^
2	^B^ 31.14 ± 0.38 ^b^	^C^ 29.59 ± 0.08 ^c^	^D^ 25.87 ± 0.15 ^bc^	^A^ 31.64 ± 0.04 ^a^
4	^A^ 27.91 ± 0.55 ^c^	^C^ 22.91 ± 0.33 ^e^	^A^ 28.18 ± 0.17 ^a^	^B^ 27.16 ± 0.11 ^b^
6	^C^ 23.05 ± 0.32 ^d^	^A^ 26.16 ± 0.58 ^d^	^D^ 21.05 ± 0.14 ^d^	^B^ 25.38 ± 0.27 ^c^
8	^D^ 23.42 ± 0.54 ^d^	^A^ 31.71 ± 0.09 ^b^	^C^ 25.39 ± 0.52 ^c^	^B^ 26.93 ± 0.08 ^b^
a*-value				
0	^C^ 17.56 ± 0.38 ^c^	^C^ 18.48 ± 1.13 ^b^	^A^ 26.77 ± 0.18 ^a^	^B^ 23.25 ± 2.39 ^bc^
2	^A^ 20.04 ± 0.12 ^b^	^B^ 19.41 ± 0.28 ^b^	^D^ 15.80 ± 0.07 ^c^	^C^ 17.44 ± 0.16 ^a^
4	^AB^ 14.91 ± 0.14 ^d^	^B^ 14.07 ± 0.76 ^d^	^A^ 16.72 ± 1.83 ^c^	^A^ 16.72 ± 0.19 ^b^
6	^C^ 17.50 ± 0.16 ^c^	^D^ 16.34 ± 0.54 ^c^	^A^ 23.37 ± 0.23 ^b^	^B^ 21.23 ± 0.17 ^d^
8	^A^ 25.39 ± 0.39 ^a^	^B^ 22.52 ± 0.30 ^a^	^B^ 22.76 ± 0.10 ^b^	^C^ 18.65 ± 0.29 ^c^
b*-value				
0	^A^ 26.09 ± 0.58 ^a^	^AB^ 24.33 ± 2.78 ^a^	^AB^ 24.40 ± 0.87 ^a^	^B^ 21.33 ± 1.48 ^a^
2	^D^ 17.54 ± 0.19 ^d^	^B^ 21.02 ± 0.10 ^bc^	^C^ 19.84 ± 0.05 ^d^	^A^ 26.90 ± 0.04 ^c^
4	^C^ 21.24 ± 0.31 ^b^	^D^ 19.38 ± 0.44 ^c^	^A^ 23.56 ± 0.25 ^b^	^B^ 22.47 ± 0.12 ^b^
6	^B^ 20.02 ± 0.36 ^c^	^A^ 22.36 ± 0.36 ^ab^	^D^ 16.52 ± 0.23 ^e^	^C^ 18.00 ± 0.43 ^c^
8	^C^ 17.53 ± 0.26 ^d^	^A^ 21.56 ± 0.17 ^bc^	^A^ 21.41 ± 0.30 ^c^	^B^ 20.63 ± 0.10 ^c^

* Values within a column (lowercase) or row (uppercase) letter are significantly different (*p* ≥ 0.05). CTS = chitosan, Nano/CTS = chitosan/nano-silica, and Nano/CTS/N = chitosan/nano-silica/nisin.

## Data Availability

Available from corresponding author.
